# Role of histone methyltransferase SETDB1 in regulation of tumourigenesis and immune response

**DOI:** 10.3389/fphar.2022.1073713

**Published:** 2022-12-13

**Authors:** Zhipeng Zhao, Lu Feng, Xuerun Peng, Tingnan Ma, Rongsheng Tong, Lei Zhong

**Affiliations:** ^1^ Department of Pharmacy, Personalized Drug Therapy Key Laboratory of Sichuan Province, Sichuan Provincial People’s Hospital, School of Medicine, University of Electronic Science and Technology of China, Chengdu, China; ^2^ Department of Emergency, Sichuan Academy of Medical Sciences & Sichuan Provincial People’s Hospital, Chengdu, China

**Keywords:** SETDB1, immune escape, immune checkpoint blockade resistance, endogenous retrovirus, epigenetic therapy

## Abstract

Epigenetic alterations are implicated in tumour immune evasion and immune checkpoint blockade (ICB) resistance. SET domain bifurcated histone methyltransferase 1 (SETDB1) is a histone lysine methyltransferase that catalyses histone H3K9 di- and tri-methylation on euchromatin, and growing evidence indicates that SETDB1 amplification and abnormal activation are significantly correlated with the unfavourable prognosis of multiple malignant tumours and contribute to tumourigenesis and progression, immune evasion and ICB resistance. The main underlying mechanism is H3K9me3 deposition by SETDB1 on tumour-suppressive genes, retrotransposons, and immune genes. SETDB1 targeting is a promising approach to cancer therapy, particularly immunotherapy, because of its regulatory effects on endogenous retroviruses. However, SETDB1-targeted therapy remains challenging due to potential side effects and the lack of antagonists with high selectivity and potency. Here, we review the role of SETDB1 in tumourigenesis and immune regulation and present the current challenges and future perspectives of SETDB1 targeted therapy.

## Introduction

Immune checkpoint blockade (ICB) therapy eradicates tumour cells *via* releasing the brake on the adaptive immune response and has revolutionized clinical treatments for multiple malignancies (Deng and Zhang, 2018; Bagchi et al., 2021). To date, 18 immune checkpoint inhibitors (ICIs) have been approved as cancer therapeutics, including 12 programmed cell death protein 1 (PD-1) monoclonal antibodies (pembrolizumab, nivolumab, cemiplimab, toripalimab, sintilimab, camrelizumab, tislelizumab, zimberelimab, penpulimab, dostarlimab, serplulimab and prolgolimab), five programmed cell death ligand 1 (PD-L1) monoclonal antibodies (atezolizumab, durvalumab, avelumab, envafolimab and sugemalimab), and one monoclonal antibody that blocks cytotoxic T-lymphocyte associated protein 4 (ipilimumab) (Dhillon, 2021; Dhillon and Duggan, 2022; Lee, 2022; Markham, 2022; Yi et al., 2022). Despite considerable advancements, the response rate to ICIs is currently limited to 10%–25% in most tumour types (Schoenfeld and Hellmann, 2020), and those with deficient immunogenic epitopes (low mutational burden) (Verdegaal et al., 2016; Tran et al., 2017), impoverished tumour-infiltrating lymphocytes (Galluzzi et al., 2018; Fanale et al., 2022), or profuse immunosuppressive factors (such as PD-L1, CD73, and indoleamine 2,3-dioxygenase 1) (Young et al., 2016; Chen and Mellman, 2017; Song et al., 2021) are less likely to respond (Galluzzi et al., 2018). Furthermore, initial ICI responders may develop acquired resistance (Schoenfeld and Hellmann, 2020). These limitations have motivated efforts to explore novel immunosuppressive mechanisms and immunotherapy approaches to complement current ICB treatments.

Epigenetic dysregulation is one of the most important hallmarks of tumourigenesis (Hanahan and Weinberg, 2011). Since accumulating evidence has identified epigenetic alterations as drivers of immune escape, epigenetic therapies could enhance response and overcome ICB resistance (Gomez et al., 2020; Topper et al., 2020). Unlike gene mutations, epigenetic modifications are heritable phenotypic alterations that do not change the nucleotide sequence and mainly include histone modification, DNA methylation and changes in chromatin structure and microRNA levels (Cheng et al., 2019; Cao and Yan, 2020). These modifications mediate cancer immunoediting, characterised by decreased antigenicity and immunogenicity, disruption of key interactions for immune response, establishment of T cell exhaustion, and creation of an immunosuppressive tumour microenvironment (TME) (Gomez et al., 2020; Gangoso et al., 2021). The reversibility and targetability of epigenetic alterations make them attractive interventions for promoting tumour cell response to immunotherapy. SET domain bifurcated histone lysine methyltransferase 1 (SETDB1) was recently identified as a critical epigenetic regulator that contributes to the immunosuppressive TME and ICB resistance (Griffin et al., 2021; Zhang et al., 2021). Here, we review the effects of SETDB1 on tumourigenesis and progression, particularly its role in regulating the tumour immune response, and present the current challenges and future perspectives of antitumour therapy targeting SETDB1.

## SETDB1 structure and regulation

SETDB1, also known as ERG-associated protein with SET domain (ESET), belongs to the histone lysine methyltransferase family. It maps to chromosome 1q21.3 and comprises 1291 amino acids with a molecular weight of 143.1 kDa (Markouli et al., 2021b). SETDB1 is highly evolutionarily conserved, and mouse SETDB1 shows 92% similarity with human SETDB1 at the amino acid level (Yang et al., 2002). SETDB1 consists of an N-terminus containing two nuclear export signal (NES) domains, two nuclear localisation signal (NLS) domains, two Tudor domains and a methyl-CpG-binding domain (MBD). The C-terminus contains the pre-SET, bifurcated SET and post-SET domains (Supplementary Figure 1A) (Batham et al., 2019; Markouli et al., 2021a). The NES and NLS domains in the N-terminus regulate SETDB1 localisation (Cho et al., 2013). The Tudor domains may anchor SETDB1 to arginine and lysine residues on histone or nonhistone substrates and are involved in forming transcriptional repression-associated multiprotein complexes, such as the Kruppel-associated box (KRAB) zinc finger protein-KRAB-associated protein-1 (KRAB-ZFP-KAP1) complex (Schultz et al., 2002). The MBD CpG domain contains two arginine residues that bind to methylated DNA (Ho et al., 2008), thereby regulating the interaction with DNA (cytosine-5) methyltransferase 3, heterochromatin formation and gene silencing by coordinating CpG methylation with H3K9 trimethylation (Li et al., 2006). The C-terminus domains of SETDB1 are responsible for its methyltransferase catalytic activity (Yang et al., 2002). The bifurcated SET domain is the main region of catalytic activity and is separated by a 347-amino acid insert. Although the exact function of this insertion remains unelucidated, SETDB1 ubiquitination at lysine 867 within the SET insertion is essential for the acquisition of complete enzymatic activity of SETDB1 in mammals (Ishimoto et al., 2016). Additionally, SETDB1 recruits S-adenosine methionine (SAM) and human homolog of murine activating transcription factor a (ATFa)-associated modulator (hAM) as cofactors during the catalytic process (Supplementary Figure S1B) (Wang et al., 2003).

SETDB1 is an epigenetic modulator that transfers methyl groups to histones and represses target gene transcription. Histones are proteins that provide structural support, around which DNA wraps to generate nucleosomes and subsequently tightly packed chromatin (Kouzarides, 2007). They can be chemically modified by some enzymes to modulate gene transcription, and methylation of lysine residues is one of the most common modifications of histones, which can affect chromatin structure and the interaction of transcription factors with nucleosomes (Hyun et al., 2017). The nucleosome is a histone octamer that consists of two duplicates of each core histones H2A, H2B, H3, and H4 (Hyun et al., 2017). SETDB1 is a histone H3 lysine 9 (H3K9) methyltransferase responsible for the chemical modifications of H3K9 di- and tri-methylation on euchromatin (Yang et al., 2002; Wang et al., 2003). This post-translational modification recruits heterochromatin protein 1 (HP1) to euchromatic promoters and establishes a heterochromatin-like silenced state at euchromatin with the coordination of KAP1 (Schultz et al., 2002; Ayyanathan et al., 2003).

## SETDB1 and tumourigenesis

**FIGURE 1:**
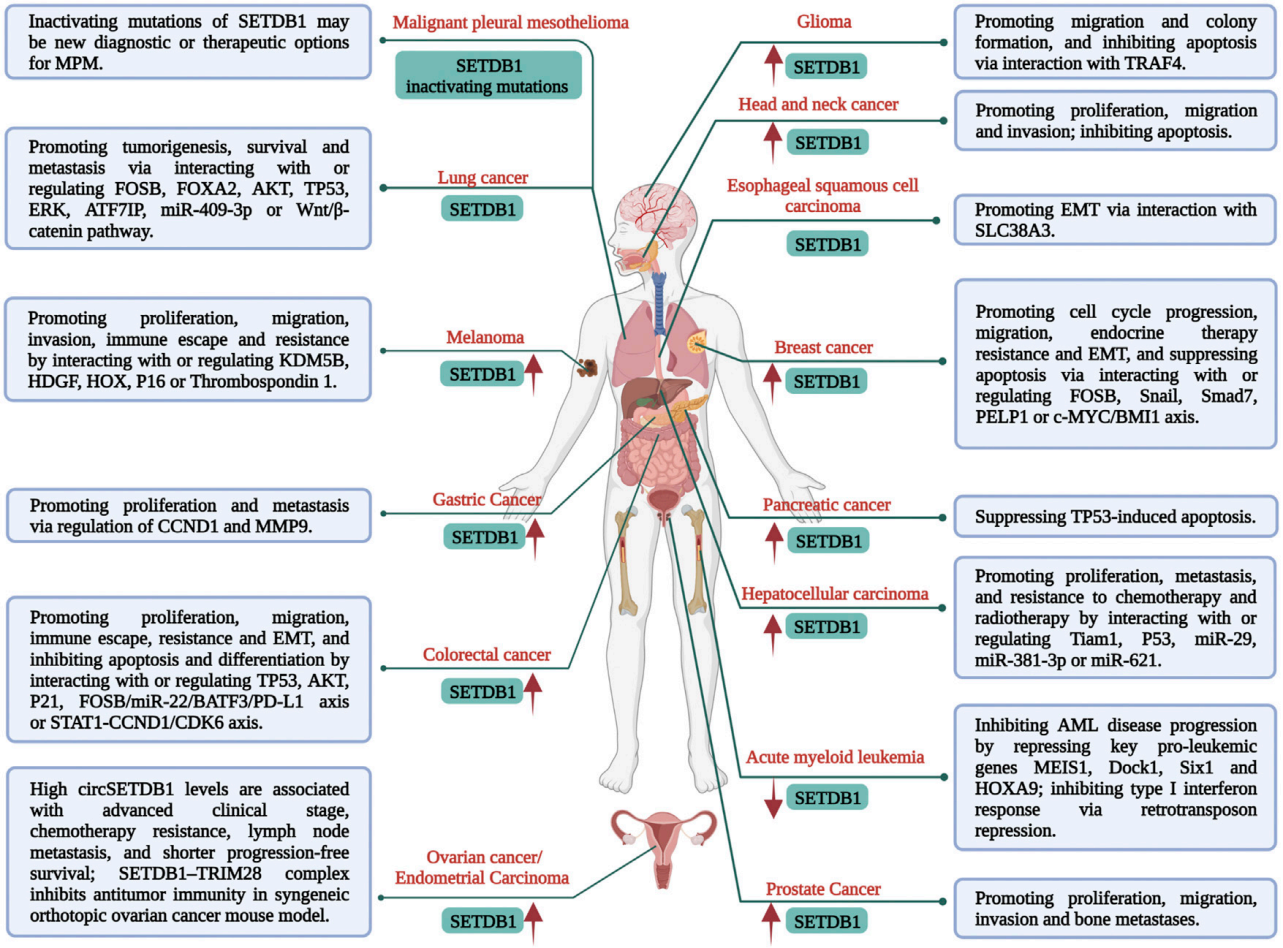
Multiple functions and mechanisms of SETDB1 regulation in malignancies.

Growing evidence has revealed the close correlation of SETDB1 overexpression and abnormal activity in a variety of malignancies with an unfavourable prognosis of cancer patients (Figure 1) (Wong et al., 2016; Orouji et al., 2019; Yu et al., 2020; Strepkos et al., 2021). Additionally, SETDB1 amplification plays pivotal roles in tumourigenesis and progression, such as promoting cell proliferation, migration, invasion, epithelial-mesenchymal transition (EMT), metastasis, resistance, and immune evasion (Figure 1). Mechanically, elevated SETDB1 facilitates H3K9 methylation at the promoter regions of multiple tumour-suppressive genes, leading to gene silencing and tumourigenesis (Cao et al., 2020; Ogawa et al., 2020; Strepkos et al., 2021). Meanwhile, various other mechanisms are also involved. For example, activated AKT kinase is beneficial for cancer cell proliferation and survival, while SETDB1 could activate AKT by mediating methylation of AKT K64 residue and promote the development of non-small-cell lung carcinoma (NSCLC) (Wang et al., 2019). Leonard I. Zon group identified SETDB1 as a cooperator of BRAF (V600E) mutation to accelerate melanoma formation (Ceol et al., 2011). In this study, SETDB1 was found to form a complex with other H3K9 methyltransferases, such as SUV39H1, and they acted together to alter gene transcription in a manner that promotes onset and invasiveness of melanoma. Moreover, SETDB1 is also considered an oncogene in hepatocellular carcinoma (HCC) cells because it boosts their proliferation and migration *via* interaction with T-cell lymphoma invasion and metastasis-inducing protein 1 (Tiam1) (Zhang et al., 2018). SETDB1 upregulation was reported to be closely related to the progression and poor prognosis of patients with HCC and was found in all metastatic foci, demonstrating the important role of SETDB1 in HCC aggressiveness. miR-29 was identified as a negative modulator of SETDB1 in this process (Wong et al., 2016).

The initial step in metastasis is EMT, which leads to a motile phenotype in cancer cells. SETDB1 was reported to participate in regulating EMT in colorectal cancer (CRC) cells by binding to the P21 promoter and affecting its activity (Cao et al., 2020), in esophageal squamous cell carcinoma *via* interaction with SLC38A3 (Liu et al., 2020), and in HCC by interacting with Tiam1 (Zhang et al., 2018). Treatment resistance in cancer may be intrinsic or acquired, which immensely restricts the efficacy of anti-cancer therapeutics. SETDB1 has also been implicated in tumour resistance. Guler et al. (2017) found elevated levels of SETDB1 in multiple drug-tolerant cancer cells, and increased H3K9me3 was enriched at long interspersed nuclear element-1 (LINE-1) genomic loci. Thus, derepression of LINE-1 elements by SETDB1 or G9a loss could restore treatment sensitivity. SETDB1 overexpression was also found to mediate the resistance of breast cancer cells to tamoxifen. This is attributed to the SETDB1 regulation on the target genes of estrogen receptor (ER) and AKT, while ER coregulator PELP1 interacts directly with SETDB1 and is necessary for SETDB1-mediated regulatory effects and tamoxifen resistance (Liu et al., 2022). Notably, although less reported, SETDB1 can also play a tumour-suppressive role, such as in acute myeloid leukaemia (AML) (Ropa et al., 2020) and some metastatic lung cancer cells (Wu et al., 2014). Thus, the role of SETDB1 in cancer may depend on its type and stage.

## SETDB1 functions in immune regulation

The regulation of immune responses mediated by SETDB1 is complex and involves multiple mechanisms. The most prominent is the limitation of endogenous retrotransposon expression, which enables tumour cells to evade innate immune sensing (Zhang et al., 2021; Pan et al., 2022). Retrotransposons are transposable elements (TEs) that include LINEs and endogenous retroviruses (ERVs). They amplify *via* reverse transcription of RNA into DNA using an RNA transposition intermediate (Wu et al., 2021). Retrotransposons only exist in eukaryotes, and over 40% of the human genome is derived from retrotransposons. Due to their viral origin, the nucleic acids that they produce are recognised as ‘non-self’ pathogen-specific molecules by the innate sensors and trigger an immune response (Tie and Rowe, 2017; Wu et al., 2021). Specifically, double-stranded RNA (dsRNA) derived from endogenous retroviral elements can be induced by low-dose 5-aza-2-deoxycytidine (a DNA methylation inhibitor), and then activate interferon through MDA5/MAVS/IRF7 sensors, thereby exhibiting anti-tumour effects (Roulois et al., 2015). Additionally, accumulation of cytoplasmic DNA resulting from retroelements or DNA damage was also reported as a pathogen-associated molecular pattern. It could be recognized by cGAS/STING signalling axis, which then activated type I interferon and promoted responses to innate immunity (Sun et al., 2013; Härtlova et al., 2015).

Cuellar et al. first reported the function of SETDB1-mediated ERV suppression in cancer cells in 2017 when they discovered that SETDB1 silencing in AML cells elevated the expression of IFN-β and IFN-stimulated genes. IFN induction by SETDB1 disruption was attributed to decreased H3K9me3 at repetitive loci, upregulation of some ERV subfamilies, LINEs and satellite repeats, and subsequent production of dsRNA that elicited a cytosolic, nucleic acid-sensing cascade and IFN-mediated cellular apoptosis (Cuellar et al., 2017). Meanwhile, Hu et al. found that activating transcription factor 7-interacting protein (Atf7ip) and its binding partner SETDB1 were chromatin modifiers that mediated tumour immune escape, and deletion of either Atf7ip or SETDB1 stimulated antigen expression and presentation, promoted T-cell activation and infiltration, resulting in augmented antitumour immune responses. In this process, SETDB1 deficiency plays an essential role in decreasing H3K9me3 deposition on the chromosomes encoding ERV-derived antigens and up-regulating tumour immunogenicity (Hu et al., 2021). Recently, two studies published in *Nature* systematically revealed the effects of and mechanisms behind SETDB1 regulation of tumour-intrinsic immunogenicity (Griffin et al., 2021; Zhang et al., 2021). They identified SETDB1 (or SETDB1 recruited by KDM5B) as a chromatin regulator that depresses tumour-intrinsic immunogenicity and mediates immune tolerance in both mice and human tumours, and SETDB1 depletion potently boosted the efficacy of ICB in mouse models. Mechanistically, SETDB1 epigenetically silences broad domains, mainly at segmental duplications-evolving loci that are enriched with TEs and immune genes. SETDB1 knockout recovers the potential of multiple TEs in encoding retroviral antigens and generating major histocompatibility complex class I (MHCI) peptides and derepresses immunostimulatory genes, thereby triggering T cell responses (Griffin et al., 2021). Moreover, SETDB1 was also found to repress the expression of radiation-induced ERVs, and SETDB1 deletion potently enhanced ERV expression and increased type I interferons, promoting antitumour immunity and sensitizing cancer radiotherapy (Pan et al., 2022).

Besides the ERV-related mechanism, SETDB1 also mediates immune response by regulating cytokine expression and some signalling axes (Hachiya et al., 2016; Lin et al., 2021). In macrophages, SETDB1 effectively inhibits Toll-like receptor 4 (TLR4)-mediated interleukin 6 (IL-6) expression by increasing H3K9 methylation levels on the IL-6 promoter and restraining its transcription. SETDB1 loss reduces H3K9me3 and enhances TLR4-mediated nuclear factor-κB (NF-κB) recruitment to the IL-6 promoter, which increases IL-6 expression *in vitro* and *in vivo* (Hachiya et al., 2016). T helper 17 (Th17) cells are implicated in multiple autoimmune diseases and cancers. OX40 inhibits Th17 cell function in a RelB (an NF-κB family member)-dependent manner, which recruits SETDB1 and G9a to the IL-17 locus to induce suppressive chromatin modifications, depressing IL-17 expression (Xiao et al., 2016). Moreover, Lin et al. (2021) reported that ablation of tripartite motif-containing 28 (TRIM28), a component of the human silencing hub (HUSH) complex, substantially boosted PD-L1 expression in ovarian cancer cells. As the catalytic subunit of HUSH, SETDB1 loss phenocopied TRIM28 silencing and elevated PD-L1 expression. Mechanistic studies revealed that SETDB1-TRIM28 suppression accelerated the formation of micronuclei in the cytoplasm and activated the cGAS-STING cascade-mediated innate immune response, thereby promoting PD-L1 expression and CD8^+^ T cell infiltration. Inconsistently, high levels of SETDB1 expression were positively related to PD-L1 expression in CRC. SETDB1 silencing markedly suppressed PD-L1 expression and boosted the cytotoxicity of T cells to CRC cells through the FOSB/miR-22/BATF3 cascade (Tian et al., 2022). The opposing expression of PD-L1 upon SETDB1 loss in these two studies may be attributed to the distinct tumour types and regulatory mechanisms.

Noteworthily, SETDB1 is also essential for the development of B cells and T cells (Pasquarella et al., 2016; Takikita et al., 2016). SETDB1 deficiency has been reported to impair B-cell development through unfolded protein response (UPR)-mediated pro-B cell apoptosis (Pasquarella et al., 2016). Mechanistically, SETDB1 functions as a repressor of specific ERV families and exogenous retroviruses in mouse B lymphocytes, such as the retrotransposon murine leukaemia virus (MLV), while SETDB1 depletion derepresses the expression of MLV proteins, leading to UPR-mediated apoptosis (Collins et al., 2015; Pasquarella et al., 2016). Likewise, impaired T-cell development was also observed in thymocyte-specific SETDB1 knockout mice, particularly CD8-lineage T cells, which exhibited increased apoptosis (Takikita et al., 2016). This is partially caused by SETDB1 deficiency-mediated derepression of FcγRIIb expression, and FcγRIIb can suppress ERK activation and induce cell apoptosis (Takikita et al., 2016). Martin et al. (2015) also reported the role of SETDB1 repression of FcγRIIb in thymocyte development. They revealed that SETDB1 deletion activated FcγRIIb expression, which then led to increased ZAP70 phosphorylation and apoptosis of CD4/CD8 double-positive T cells. Additionally, Jarid2 directly interacts with SETDB1 and recruits it to the *Zbtb16* locus that encodes promyelocytic leukemia zinc finger (PLZF) protein, a signature transcription factor of immature invariant natural killer T (iNKT) cells, and its deficiency in thymocytes will affect the proper development of iNKT cells through decreasing H3K9me3 and upregulating PLZF levels (Pereira et al., 2014). These findings reveal the essential role of SETDB1 in immune cell development, which cannot be ignored in SETDB1 targeted therapy.

## Overview of pharmacologic SETDB1 inhibitors

Given the important role of SETDB1 in tumourigenesis, progression, and tumour immune escape, developing SETDB1 antagonists is a promising strategy for cancer chemotherapy and immunotherapy. However, there are not so many studies concerning SETDB1 antagonists at present, particularly the specific SETDB1 inhibitors. This is due to the lack of SET domain crystal structure, which is challenged by its bifurcated characteristic. Several reported SETDB1 selective inhibitors do not directly target the SET domain and are still in the stage of biochemical activity testing (Table 1). Park et al. (2017) screened peptide-competitive small molecule SETDB1 inhibitors using an approach of pharmacophore model-based virtual screening combined with biological evaluation. Two compounds VH01 and VH06 were identified as the final hits that obviously reduced H3K9me3 levels and had neuronal effects. In the surface plasmon resonance assay, VH01 and VH06 bound to SETDB1 with the K_D_ values of 3.26 ± 1.71 μM and 0.232 ± 0.146 μM, respectively. The tandem Tudor domain (TTD) of SETDB1 can recognize methylated lysine. Specifically intercepting the interaction of SETDB1-TTD with the endogenous binders is an attractive approach for the development of small molecule SETDB1 antagonists (Mader et al., 2019; Guo et al., 2021). (R, R)-59 was a selective small-molecule SETDB1-TTD inhibitor screened based on this concept, which exhibited a K_D_ value of 0.088 ± 0.045 μM in the isothermal titration calorimetry (ITC) detection (Guo et al., 2021). In addition, 5-allyloxy-2-(pyrrolidin-1-yl) quinoline (APQ) was also identified as a negative regulator of SETDB1 activity. It effectively decreased H3K9me3 levels and ameliorated Huntington’s disease symptoms *in vitro* and *in vivo* (Hwang et al., 2021).

**TABLE 1:** List of the reported small molecule SETDB1 antagonists.

Agents	Classification	Disease types	Specificity	IC_50_/K_D_	Effects	References
VH01 and VH06	Peptide-competitive SETDB1 inhibitors	Huntington’s disease	Specific	K_D_ values in SPR assay: 3.26 ± 1.71 μM for VH01; 0.232 ± 0.146 μM for VH06	Decreasing H3K9me3 level and showing neuronal effects without cytotoxicity	Park et al. (2017)
(R, R)-59	SETDB1-TTD inhibitor	Acute monocytic leukemia	Specific	K_D_ value in ITC assay: 0.088 ± 0.045 μM	Inhibiting the interaction of H3 peptide with SETDB1 tandem Tudor domain	Guo et al. (2021)
5-allyloxy-2-(pyrrolidin-1-yl) quinoline	SETDB1 inhibitor	Huntington’s disease	Specific	IC_50_: 65 μΜ in enzyme inhibition assay	Inhibiting SETDB1 activity and H3K9me3 level, and improving motor function and neuropathological symptoms with minimal toxicity in mouse HD models	Hwang et al. (2021)
Arsenic trioxide	PML-RARα fusion protein inhibitor	Acute promyelocytic leukemia	Nonspecific	—	Decreasing SETDB1 expression and increasing Id2 expression *via* inducing promyelocytic leukemia protein degradation	Cho et al. (2011)
BIX-01294	G9a HMTase inhibitor	Melanoma	Nonspecific	IC_50_s in SK-HI-SETDB1 cell line: 1.55 × 10^-9^ M at day 4 and 1.16 × 10^-9^ M at day 5	Decreasing SETDB1 activity and enhancing the efficacy of combined BRAF and MEK inhibitors (vemurafenib and trametinib) in BRAF mutant cells, as well as overcoming drug resistance	Orouji et al. (2019)
Cardamonin	NF-κB inhibitor	Breast cancer	Nonspecific	—	Suppressing the expression of stem cell-associated histone modifiers including SETDB1 and the enrichment of breast cancer stem cells, enhancing the efficacy of chemotherapeutic drugs	Jia et al. (2016)
DZNep	SAM hydrolase inhibitor	Lung cancer	Nonspecific	—	Suppressing SETDB1 and EZH2 expression, and follow-up H3K9me3 and H3K27me3 levels, resulting in induction of cell apoptosis and inhibition of cell growth	Lee and Kim, (2013)
Mithramycin A	SP1 inhibitor	Melanoma	Nonspecific	IC_50_s in SK-HI-SETDB1 cell line: 912.9 nM, 43.72 nM, and 29.06 nM after 24, 48, and 72 h, respectively	Repressing SETDB1 expression *via* blocking the interactions between SETDB1 and SP1; inhibiting cell viability, migration, and invasion; enhancing the efficacy of mitogen-activated protein kinase (MAPK) inhibitor-based therapies	Federico et al. (2020)
EC-8042	SP1 inhibitor	Melanoma	Nonspecific	—	Repressing SETDB1 expression and inducing changes at the transcriptomic, morphological, and functional level; enhancing the efficacy of MAPK inhibitor	Federico et al. (2020)
Piperlongumine	ROS inducer	Breast cancer	Nonspecific	—	Downregulating SETDB1 expression and activating FOSB expression, thereby inducing cancer cell death	Park et al. (2019)
Paclitaxel	Mitotic inhibitor	Lung cancer	Nonspecific	—	Repressing SETDB1 expression in a P53-dependent manner and upregulating FOSB, and inducing cell death *via* G2/M phase arrest	Na et al. (2016); Noh et al. (2014)
Cisplatin	Alkylating agent	Lung cancer	Nonspecific	—	Decreasing SEDB1 expression and derepressing the expression of FOSB.	Na et al. (2016)
Doxorubicin	Topoisomerase inhibitor	Lung cancer	Nonspecific	—	Decreasing SEDB1 expression and derepressing the expression of FOSB.	Na et al. (2016); Na and Kim, (2018)
5-fluorouracil	Nucleoside Antimetabolite	Lung cancer	Nonspecific	—	Decreasing SEDB1 expression and derepressing the expression of FOSB.	Na et al. (2016)

Recently, most available SETDB1 inhibitors are non-specific (Table 1), and they affect SETDB1 function by inhibiting SETDB1 expression or histone methyltransferase (HMTase) activity. Arsenic trioxide (As_2_O_3_) showed inhibitory effect on SETDB1 signalling through inducing the degradation of promyelocytic leukemia protein (Cho et al., 2011). BIX-01294 (CAS 935693-62-2), a G9a HMTase inhibitor, also had the ability to block SETDB1 activity. The combination of BIX-01294 with BRAF and MEK inhibitors exhibited a high level of synergistic effects in killing melanoma cells and even resistant cells (Orouji et al., 2019). Cardamonin could suppress the expression of multiple stem cell-associated histone modifying genes including SETDB1, and prevent the enrichment of breast cancer stem-like cells when combined with chemotherapeutic drugs (Jia et al., 2016). DZNep, a SAM hydrolase inhibitor, efficaciously depressed H3K9me3 and H3K27me3 expression through downregulation of SETDB1 and EZH2 expression. The inhibition of DZNep against multiple HMTases contributed to its anti-proliferative and pro-apoptotic activity on lung cancer cells (Lee and Kim, 2013). Mithramycin A and mithramycin analog EC-8042 were reported to inhibit SETDB1 expression and cause changes at transcriptomic and functional levels. They could sensitize melanoma cells to mitogen-activated protein kinase inhibitor (Ryu et al., 2006; Federico et al., 2020). Piperlongumine (PL), a natural alkaloid compound, was also reported to be potent in downregulating SETDB1 expression in breast cancer cells. Reduced SETDB1 induced caspase 9-dependent PARP cleavage and FOSB expression, which mediated the anti-breast cancer effects of PL (Park et al., 2019). Additionally, several chemotherapeutic agents, including paclitaxel (PTX), cisplatin, doxorubicin, and 5-fluorouracil, were identified as potential antagonists against SETDB1 expression at both transcription and protein levels (Noh et al., 2014; Na et al., 2016; Na and Kim, 2018). Of these, PTX repressed SETDB1 expression in a p53-dependent manner, and PTX-induced p53 expression might involve in heterochromatic suppression on SETDB1 promoter (Noh et al., 2014).

## Conclusion

Overall, SETDB1 amplification is closely correlated with poor prognosis of multiple malignancies and contributes to tumourigenesis, tumour progression and immune evasion. This is mainly attributed to H3K9me3 deposition by SETDB1 on tumour-suppressive genes, retrotransposons, and immune clusters. SETDB1 depletion in mouse tumours derepresses immunostimulatory genes and TE-encoded retroviral antigens and MHCI peptides, which will increase CD8^+^ T cells and p15E-specific CD8^+^ T cells expressing canonical cytotoxicity genes, triggering cytotoxic T cell responses and sensitizing tumours to ICB (Griffin et al., 2021). Therefore, SETDB1 targeting is an attractive strategy for cancer therapy, particularly in combination with immunotherapy and radiotherapy, because of its regulatory effects on ERVs. Nevertheless, SETDB1-targeted therapy remains challenging.

First, various SETDB1-associated regulatory mechanisms remain unelucidated. As already mentioned, SETDB1 is not positively associated with tumourigenesis and progression of all malignancies, and it may act as a tumour suppressor in certain tumour types or at certain stages. Meanwhile, SETDB1 is essential for immune cell development and mammalian genome stability, and activated ERVs caused by SETDB1 deletion in somatic cells may provoke carcinogenic mutations through ERV insertions. These challenges highlight the need to elucidate the differences and specific mechanisms of SETDB1 interaction with relevant target proteins, ERVs and immune genes across different tumour types and noncancerous cells. Second, SETDB1-targeted therapy is hampered by the lack of SETDB1 inhibitors with high selectivity and activity. The recently reported potent SETDB1 antagonists are mostly non-specific, and since most are cytotoxic chemotherapy drugs, off-target effects and side effects are inevitable. A potential approach to develop selective SETDB1 inhibitors is the specific blocking of SETDB1-TTD interaction. Alternatively, SETDB1 activity could be depressed by the development of selective antagonists against SAM (a methyl donor for SETDB1-mediated methylation). Moreover, the elucidation of SETDB1 regulation-related molecular mechanisms and specific targets, as well as the resolution of SET domain crystal structure will benefit the future development of therapeutics targeting SETDB1.

In summary, SETDB1 is considered an oncogene in multiple malignant tumours. Although challenged by its functions in normal cells and potential side effects, increasing evidence nevertheless strongly supports SETDB1 as a candidate target for cancer therapy, particularly immunotherapy. A deeper understanding of the molecular mechanisms of SETDB1 that regulate ERVs and immune genes in both tumour and normal cells and the availability of more selective and effective SETDB1 inhibitors may lead to SETDB1 targeting as a promising antitumour epigenetic therapy.
