# Supplementary figures and images for: Role of histone methyltransferase SETDB1 in regulation of tumourigenesis and immune response
Source: Front Pharmacol. 2022 Dec 13;13:1073713. doi: 10.3389/fphar.2022.1073713 (PMC9793902; doi:10.3389/fphar.2022.1073713)

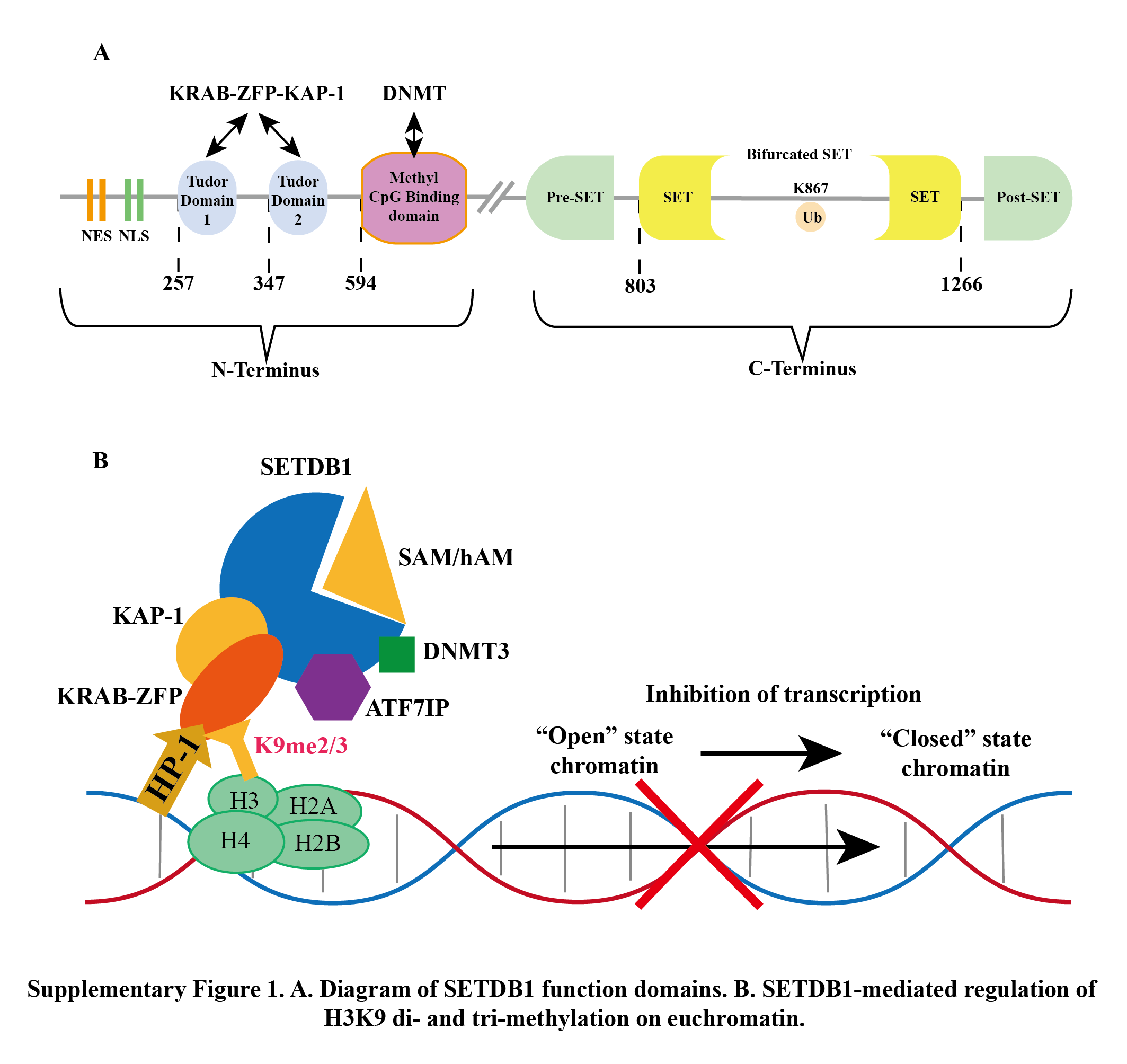

Supplement: Supplementary file 1 [file Image1.TIF]
